# Population genetic structure of the globally introduced big‐headed ant in Taiwan

**DOI:** 10.1002/ece3.9660

**Published:** 2022-12-23

**Authors:** Kuan‐Ling Liu, Shu‐Ping Tseng, Haruki Tatsuta, Kazuki Tsuji, Jia‐Wei Tay, G. Veera Singham, Chin‐Cheng Scotty Yang, Kok‐Boon Neoh

**Affiliations:** ^1^ Department of Entomology National Chung Hsing University Taichung Taiwan; ^2^ Department of Entomology National Taiwan University Taipei Taiwan; ^3^ Graduate School of Systems Life Sciences Kyushu University Fukuoka Japan; ^4^ Department of Subtropical Agro‐Environmental Sciences University of the Ryukyus Okinawa Japan; ^5^ Department of Plant and Environmental Protection Sciences University of Hawaii at Manoa Honolulu Hawaii USA; ^6^ Centre for Chemical Biology Universiti Sains Malaysia Penang Malaysia; ^7^ Department of Entomology Virginia Polytechnic Institute and State University Blacksburg Virginia USA

**Keywords:** bridgehead effect, genetic bottleneck, invasion biology, invasive ant, population structure

## Abstract

Global commerce and transportation facilitate the spread of invasive species. The African big‐headed ant, *Pheidole megacephala* (Fabricius), has achieved worldwide distribution through globalization. Since the late 19th century, Taiwan has served as a major seaport because of its strategic location. The population genetic structure of *P. megacephala* in Taiwan is likely to be shaped by international trade and migration between neighboring islands. In this study, we investigated the population genetics of *P. megacephala* colonies sampled from four geographical regions in Taiwan and elucidated the population genetic structures of *P. megacephala* sampled from Taiwan, Okinawa, and Hawaii. We observed a low genetic diversity of *P. megacephala* across regions in Taiwan. Moreover, we noted low regional genetic differentiation and did not observe isolation by distance, implying that long‐distance jump dispersal might have played a crucial role in the spread of *P. megacephala*. We sequenced the partial cytochrome oxidase I gene and observed three mitochondrial haplotypes (TW1–TW3). TW1 and TW3 most likely originated from populations within the species' known invasive range, suggesting that secondary introduction is the predominant mode of introduction for this invasive ant. TW2 represents a novel haplotype that was previously unreported in other regions. *P. megacephala* populations from Taiwan, Okinawa, and Hawaii exhibited remarkable genetic similarity, which may reflect their relative geographic proximity and the historical connectedness of the Asia‐Pacific region.

## Graphical abstract

*Pheidole megacephala* exhibits low genetic diversity across regions in Taiwan. Significant yet low regional genetic differentiation and the absence of isolation‐by‐distance implied that long‐distance jump dispersal may be relatively prevalent. Secondary introduction is the prevailing mode of introduction for *P. megacephala* and populations from Taiwan, Okinawa, and Hawaii exhibit remarkable genetic similarity.

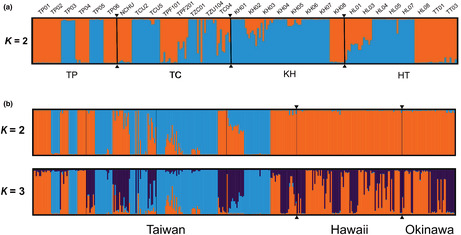

## 1 INTRODUCTION

Numerous case studies have reported that newly introduced ant species become established invaders outside their native ranges, even if they experience a substantial genetic bottleneck during colonization, which may hinder their fitness (Suarez & Tsutsui, 2008; Tsutsui et al., 2000). This characteristic suggests that the consequences of reduced genetic diversity are not always negative. The apparent paradox is that the absence of intraspecific aggression between nests leads to unicoloniality, which contributes to ecological dominance within its introduced range (Drescher et al., 2010; Hoffmann, 2014; Tsutsui et al., 2000). Furthermore, inbreeding in unicolonial invasive ants may purge deleterious alleles, enabling colonies to have an improved chance of survival in new environments (Eyer et al., 2018).

Globalization has facilitated human‐mediated biological invasion (Hulme, 2009; Meyerson & Mooney, 2007). With the expansion of international trade and advancements in transportation, the introduction of invasive species to new locations has become increasingly common. This phenomenon is termed the “bridgehead effect” or “secondary introduction” and occurs when one invasion engenders another invasion (Bertelsmeier et al., 2018; Ficetola et al., 2008; Garnas et al., 2016; Lombaert et al., 2010). This process is facilitated by the rapid evolution of invasion‐associated traits, including highly plastic life history traits and reduced inbreeding pressure in the intermediate regions, which may increase the propagule pressure of an invader in their introduced regions (Lee, Weng, et al., 2020). The development of high‐resolution genetic and genomic markers has facilitated comprehensive analyses of invasion history; such analyses have demonstrated that secondary introduction is the primary mode of introduction for numerous global invasive species, including ants (Ascunce et al., 2011; Blumenfeld et al., 2021; Sherpa et al., 2017).

The African big‐headed ant (*Pheidole megacephala*) is an invasive ant species native to Africa and was introduced to most of the world's temperate and tropical zones (Wetterer, 2012). Similar to most invasive ant species (Hee et al., 2000; Tay et al., 2014), *P. megacephala* may not require a large propagule size for successful establishment because a founding propagule comprising 1 queen and at least 10 workers or pupae is sufficient to ensure colony survival (Chang, 1985). *P. megacephala* are habitat generalists, preferring to nest in soil and dead tree logs, which enables them to be transported readily with exported commodities and logs or timber (Sarnat et al., 2015). *P. megacephala* colonies are ecologically and competitively dominant in most areas to which they are introduced, displacing native vertebrates and invertebrates (Burwell et al., 2012; Callan & Majer, 2009; Dejean et al., 2007, 2008; Hoffmann et al., 1999; Plentovich et al., 2009; Strohecker, 2012; Vanderwoude et al., 2000). In Taiwan, the dominance of *P. megacephala* found in the forest edge has substantially contributed to the collapse of the ant community and species interaction network in the forest (Tsai, 2019). The competitive exclusion of other ants from the forest may decrease the forest interior by at least 1 km from its edge (Tsai, 2019).

In the late 19th century, *P. megacephala* was documented in Africa, the Indian Ocean islands, the Atlantic islands, East Asia, Australia, Hawaii, South America, Central America, and the West Indies (Wetterer, 2012). By the 20th century, this ant species had spread to numerous nearby islands in the Pacific region, such as Hawaii and Australia (Wetterer, 2007). Although their presence in Asia‐Pacific countries was documented, these ants' phylogenetics and population genetics received little attention.

To fill the aforementioned research gap, the present study used mitochondrial DNA (mtDNA) and microsatellite DNA to examine the population genetic structure of *P. megacephala* in Taiwan in order to understand demographic events (e.g., genetic bottleneck and rapid expansion) after their invasion. We inferred the ant population dynamics and postinvasion dispersal pattern in Taiwan. Moreover, we determined whether *P. megacephala* populations in Taiwan share a common origin with one or more native/introduced populations that share the same patterns as populations in the United States and Australia. Conversely, *P. megacephala* populations may have arisen multiple times from an unidentified origin, considering that research on the phylogenetics of the test species is limited. Accordingly, we investigated the genetic relationships among the *P. megacephala* populations from Taiwan, those from two Pacific islands, and those with genetic data on GenBank (including native and several selected introduced ranges).

## 2 MATERIALS AND METHODS

### 2.1 Sample collection

**FIGURE 1:**
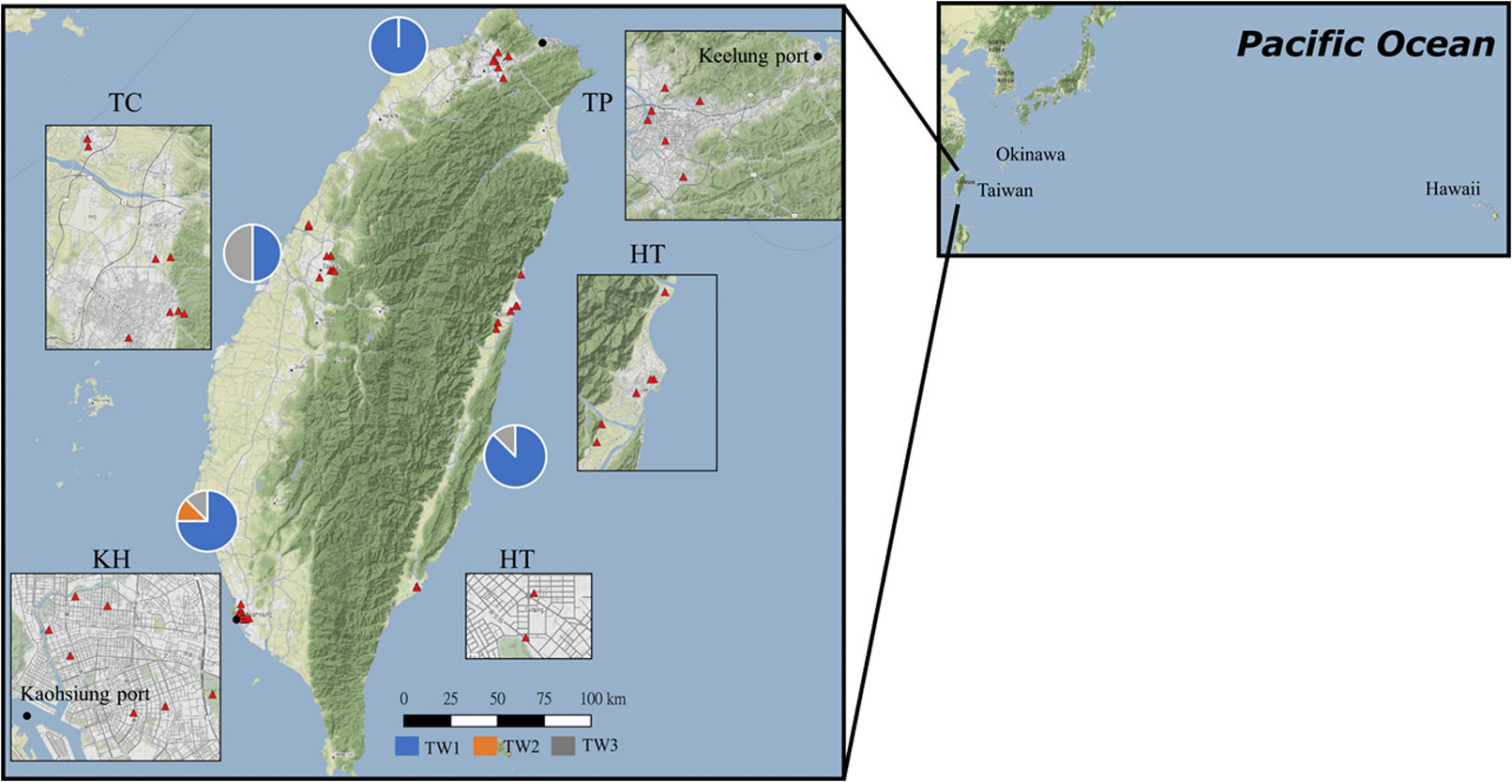
Locations of 30 colonies sampled from four administrative regions in Taiwan (red triangle). Black circles indicate Kaohsiung and Keelung, the two major seaports constructed in 1858 and 1913, respectively. They have remained Taiwan's international commerce centers. All three haplotypes with different frequencies were recovered in the sampling regions. Populations sampled from Okinawa and Hawaii were also included for analysis because they are probably historically linked to the populations in Taiwan.

A total of 30 *P. megacephala* colonies were sampled from urban parks in four Taiwanese regions (six parks in Taipei [TP], eight in Taichung [TC], eight in Kaohsiung [KH], and eight in Hualien and Taitung [HT]; Figure 1, Appendix S1) during 2018–2019. Workers collected from each colony were transported to the laboratory for aggression tests to ensure that they originated from different colonies. Our preliminary study revealed that colonies located more than 100 m apart acted aggressively. Furthermore, 6 and 13 colonies of this species were collected from Okinawa and Hawaii, respectively (Appendix S1). All samples used in this study were minor workers. The workers were preserved in 95% alcohol and refrigerated at 4°C until DNA extraction. Morphological identification was based on the methods of Bolton (1994), Lin (1998), and Sarnat et al. (2015). Our preliminary result indicated no cryptic species in our sampling areas (Liu, 2020; Appendix S2). This finding supports that of Wills et al. (2014), who reported that *P. megacephala* was a single species within its exotic range.

### 2.2 Molecular techniques

Genomic DNA was extracted from eight workers from each colony by using the Gentra Puregene Tissue kit (Qiagen) and stored at 4°C for subsequent genetic analyses. The DNA of these eight workers from each colony was used for sequencing and genotyping. The partial cytochrome oxidase I gene (*COI*) sequences, commonly used in DNA barcoding, were amplified using the primers LCO1490 (5′‐GTCAACAAATCATAAA GATATTGG‐3′) and HCO2198 (5′‐TAAACTTCAGGGTGACCAAAAAATCA‐3′) to target a 708 bp fragment (Folmer et al., 1994). Polymerase chain reaction (PCR) was performed in a 25 μl reaction tube containing 2 μl of template DNA (25–50 ng), 12.5 μl of TaKaRa EmeraldAmp Max PCR Master Mix (TaKaRa), 0.2 μM forward and reverse primers, and ddH_2_O. The thermocycling conditions were as follows: initial denaturation at 94°C for 3 min, followed by 35 cycles, each consisting of 94°C for 30 s, 55°C for 30 s, and 72°C for 40 s, and a final step at 72°C for 10 min. PCR products were cleaned using Zymo DNA Clean and Concentrator‐5 Kit (Zymo Research), and were subjected to Sanger sequencing in both the forward and reverse directions.

Eight workers from each colony were genotyped, resulting in a total of 384 individuals, at seven dinucleotide‐repeat microsatellite loci: *Pmeg‐06*, *Pmeg‐07*, *Pmeg‐09*, *Pmeg‐10*, *Pmeg‐11*, *Pmeg‐12*, and *Pmeg‐14* (Fournier et al., 2008). The PCR multiplex reactions were divided into two groups. The first group (*Pmeg‐06*, *Pmeg‐09*, *Pmeg‐12*, and *Pmeg‐14*) was subjected to the following thermocycling conditions: initial denaturation at 95°C for 15 min, followed by 35 cycles, each consisting of 94°C for 30 s, 60°C for 90 s, and 72°C for 60 s, and a final step at 60°C for 30 min. The second group (*Pmeg‐07*, *Pmeg‐10*, and *Pmeg‐11*) was subjected to the following thermocycling conditions: initial denaturation at 95°C for 15 min, followed by 35 cycles, each consisting of 94°C for 30 s, 56°C for 90 s, and 72°C for 60 s, and a final step at 60°C for 30 min. All PCR products were analyzed using the ABI 3730XL DNA Analyzer (Applied Biosystems) by Genomics BioSci and Tech (Taipei); GeneMarker (version 2.6.0; SoftGenetics LLC) was used to visualize and score alleles.

### 2.3 Microsatellite genetic analyses in the Taiwanese population

GenAlEx (version 6.5; Peakall & Smouse, 2006) was used to evaluate the allele frequency, number of alleles *N*_A_, expected heterozygosity *H*_E_, and observed heterozygosity *H*_O_ for every locus and region. The allelic richness *A*r was calculated using FSTAT (Goudet, 2001). The genetic diversity of microsatellite loci was compared using analysis of variance conducted on SPSS (version 11.0; SPSS) followed by Tukey's honestly significant difference post hoc test for multiple comparisons (*α* = .05); *N*_A_ was compared using the Kruskal–Wallis test.

BOTTLENECK (Cornuet & Luikart, 1996; Piry et al., 1999) was employed to determine whether the population experienced a drastic reduction in genetic diversity by using distinct mutation models: a two‐phase model (TPM) with 90% single‐step mutations and another TPM with 10% multistep mutations. Significance was tested using a Wilcoxon signed‐rank test as recommended by Piry et al. (1999) because the number of loci we used was <20. The occurrence of a bottleneck event was determined using the mode‐shift test, which reveals the allele frequency distribution. Isolation by distance was determined by plotting the [*F*_ST_/(1 − *F*_ST_)] coefficients and the logarithm of the geographical distance. Isolation by distance was tested at among‐colony and among‐region levels. The significance of the correlation was tested using the Mantel test in GENEPOP (version 4.5; Raymond & Rousset, 1995).

Hierarchical analysis of molecular variance (AMOVA; Excoffier et al., 1992), implemented in GenAlEx (version 6.5; Peakall & Smouse, 2006), was used to determine variances at three levels: variances among the four geographical regions, variances among colonies, and variances between samples within colonies. The fixation indices for pairwise comparisons were determined using 999 permutations (Nei, 1973).

### 2.4 Genetic differentiation among the three islands and within Taiwan

Genetic differentiation among Hawaii, Okinawa, and Taiwan, in addition to that among Taiwan's four regions, was estimated using *F*_ST_, and the corresponding significance was tested using a permutation test on GENEPOP (version 4.5; Raymond & Rousset, 1995). To investigate the population structures in Hawaii, Okinawa, and Taiwan, in addition to that in Taiwan's four regions, we used the Bayesian clustering method‐based program STRUCTURE (version 2.3.4; Pritchard et al., 2000). All analyses were performed under an admixture model by using a Markov chain Monte Carlo (MCMC) run for 1 million generations with a 100,000 burn‐in for cluster sizes ranging from 1 to 10 (i.e., *K* = 1–10). Each *K* was tested 10 times. Structure Harvester (Earl & Vonholdt, 2012) was used to determine the best score for *K* values supported by the Delta *K* method (Evanno et al., 2005). The structure results were summarized and used to generate graphs through the CLUMPAK server (http://clumpak.tau.ac.il; Jakobsson & Rosenberg, 2007). Principal component analysis (PCA) was used to visualize the population structure by plotting individual data in the R package adegenet (Jombart, 2008; R Core Team, 2020).

### 2.5 mtDNA analyses

Sequences from both ends were manually edited and aligned using the ClustalW algorithm implemented in Bioedit (Hall, 2011) and assessed in MEGA (version 7.0; Kumar et al., 2016). Several *COI* sequences of *P. megacephala* from multiple studies (Fournier et al., 2012; Kartzinel & Pringle, 2015; Moreau, 2008; Smith & Fisher, 2009; Wills et al., 2014) were included in mtDNA analyses. Some of these mtDNA sequences do not share the exact genomic region amplified by the primers used in the present study; hence, only 310 bp fragments of these sequences that partially overlapped with our sequence were used in the subsequent analysis (Appendix S5). A phylogenetic tree based on truncated sequences was constructed using MrBayes (version 3.2; Ronquist et al., 2012) by selecting the generalized time‐reversible model with gamma‐distributed rate variation across sites and a proportion of invariable sites as the evolutionary model. Two parallel MCMC simulations were run for 2 × 10^6^ generations by using four chains (three heated and one cold), with each run sampling every 500 generations. A rapid bootstrap analysis and a search for the best‐scoring maximum‐likelihood tree were conducted using the extended majority rule‐based bootstrapping criterion (Pattengale et al., 2010). All results were obtained using the general time‐reversible nucleotide substitution model. *Pheidole sexspinosa* Mayr and *P. xerophila* (Fournier et al., 2012; Wheeler & Sauter, 1909) were included as outgroup species (Appendix S3).

## 3 RESULTS

### 3.1 Genetic diversity and bottleneck in the Taiwanese population

Two to six alleles were identified across seven polymorphic microsatellite loci (Table 1). A total of 12 private alleles across 6 of 7 microsatellite loci were identified. The average private allele frequencies were 0.049, 0.013, 0.008, and 0.035 in TP, TC, KH, and HT, respectively. The mean ± standard error for *N*_A_, *A*r, *H*_E_, and *H*_O_ in Taiwan were 3.857 ± 0.234, 2.615 ± 0.149, 0.512 ± 0.028, and 0.272 ± 0.029, respectively. No significant differences in *N*_A_, *A*r, *H*_E_, or *H*_O_ were observed between the four regions (*p* = .509, .473, .351, and .148, respectively).

**TABLE 1:** Genetic diversity in seven microsatellite loci of *Pheidole megacephala* in Taiwan.

Locus	TP (*n* = 48)				TC (*n* = 64)				KH (*n* = 64)				HT (*n* = 64)			
	*N*_A_	*A*r	*H*_E_	*H*_O_	*N*_A_	*A*r	*H*_E_	*H*_O_	*N*_A_	*A*r	*H*_E_	*H*_O_	*N*_A_	*A*r	*H*_E_	*H*_O_
Pmeg06	3.000	3.000	0.493	0.214	4.000	2.750	0.599	0.295	5.000	3.693	0.622	0.232	4.000	2.750	0.637	0.377
Pmeg07	4.000	3.000	0.663	0.479	4.000	3.985	0.675	0.323	5.000	2.999	0.656	0.172	4.000	3.749	0.633	0.563
Pmeg09	5.000	3.000	0.566	0.313	3.000	2.000	0.516	0.456	3.000	2.950	0.588	0.413	3.000	2.000	0.467	0.492
Pmeg10	3.000	2.000	0.465	0.042	4.000	2.000	0.503	0.188	2.000	1.950	0.253	0.047	5.000	3.500	0.612	0.286
Pmeg11	2.000	2.000	0.444	0.000	2.000	1.993	0.371	0.230	4.000	1.000	0.119	0.094	4.000	2.000	0.522	0.094
Pmeg12	4.000	2.000	0.490	0.104	6.000	3.699	0.668	0.397	6.000	2.000	0.487	0.234	6.000	4.250	0.684	0.323
Pmeg14	2.000	2.000	0.153	0.125	5.000	1.999	0.443	0.311	2.000	2.000	0.492	0.469	4.000	2.943	0.519	0.339
Mean	3.286	2.430	0.468	0.182	4.000	2.632	0.540	0.314	3.857	2.370	0.460	0.237	4.286	3.027	0.582	0.353

Abbreviations: *A*r, allelic richness; *H*_E_, expected heterozygosity; *H*_O_, observed heterozygosity; *n*, total number of colonies; *N*_A_, number of alleles.

**FIGURE 2:**
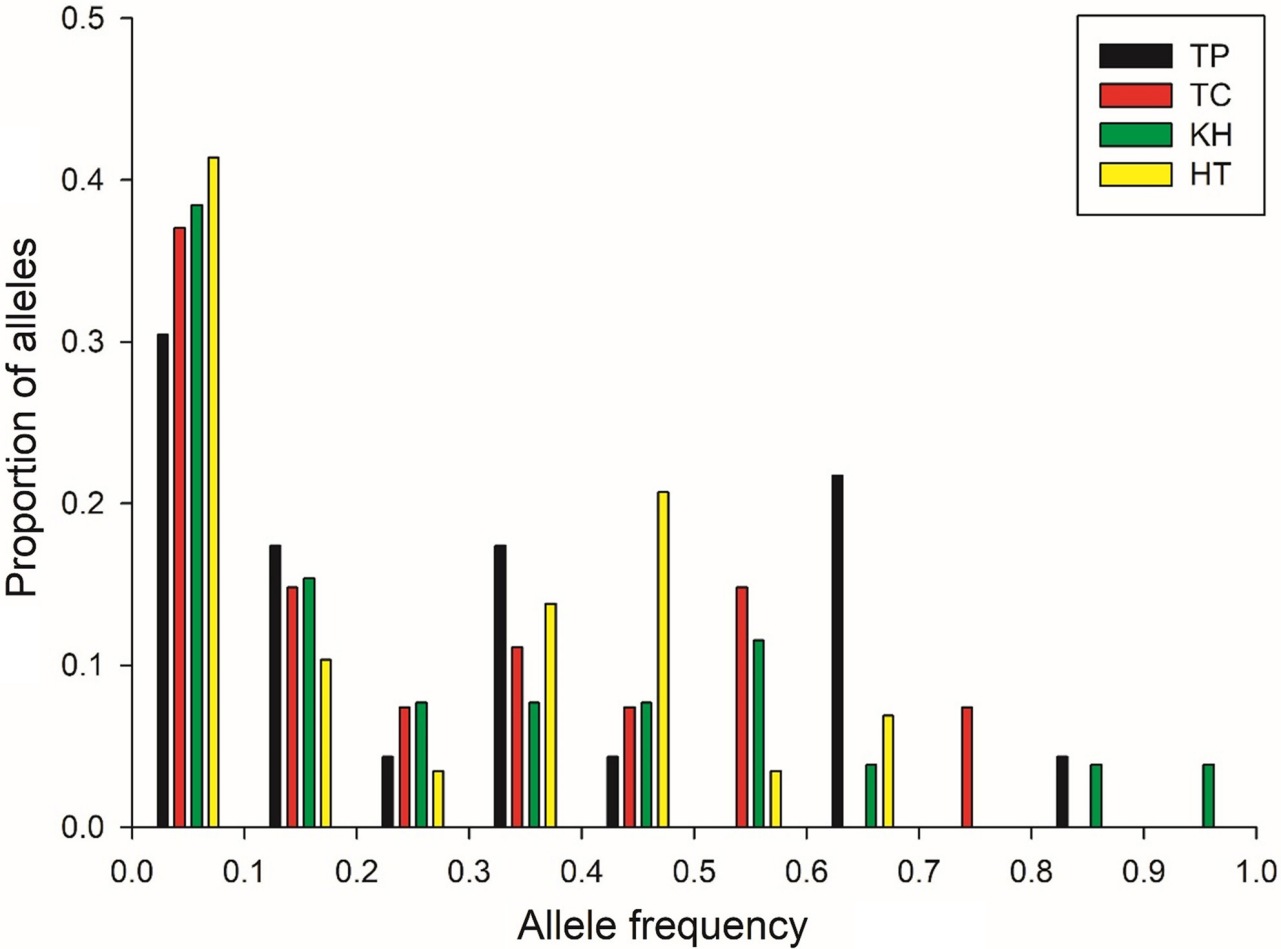
Allele frequency distribution at seven microsatellite loci in ants sampled from four regions.

No significant heterozygosity excess was observed in all regions under the TPM model (Appendix S4 and Table S1). The allele frequency distribution revealed a high proportion of low‐frequency alleles, resulting in a normal L‐shaped curve in the mode‐shift test (Figure 2). Because of the small sample size, a microsatellite data‐fitting TPM model was used (Di Rienzo et al., 1994; Piry et al., 1999). The Wilcoxon signed‐rank and mode‐shift tests revealed that the populations had not experienced a recent bottleneck.

### 3.2 Genetic differentiation and population structure within the Taiwanese population

**FIGURE 3:**
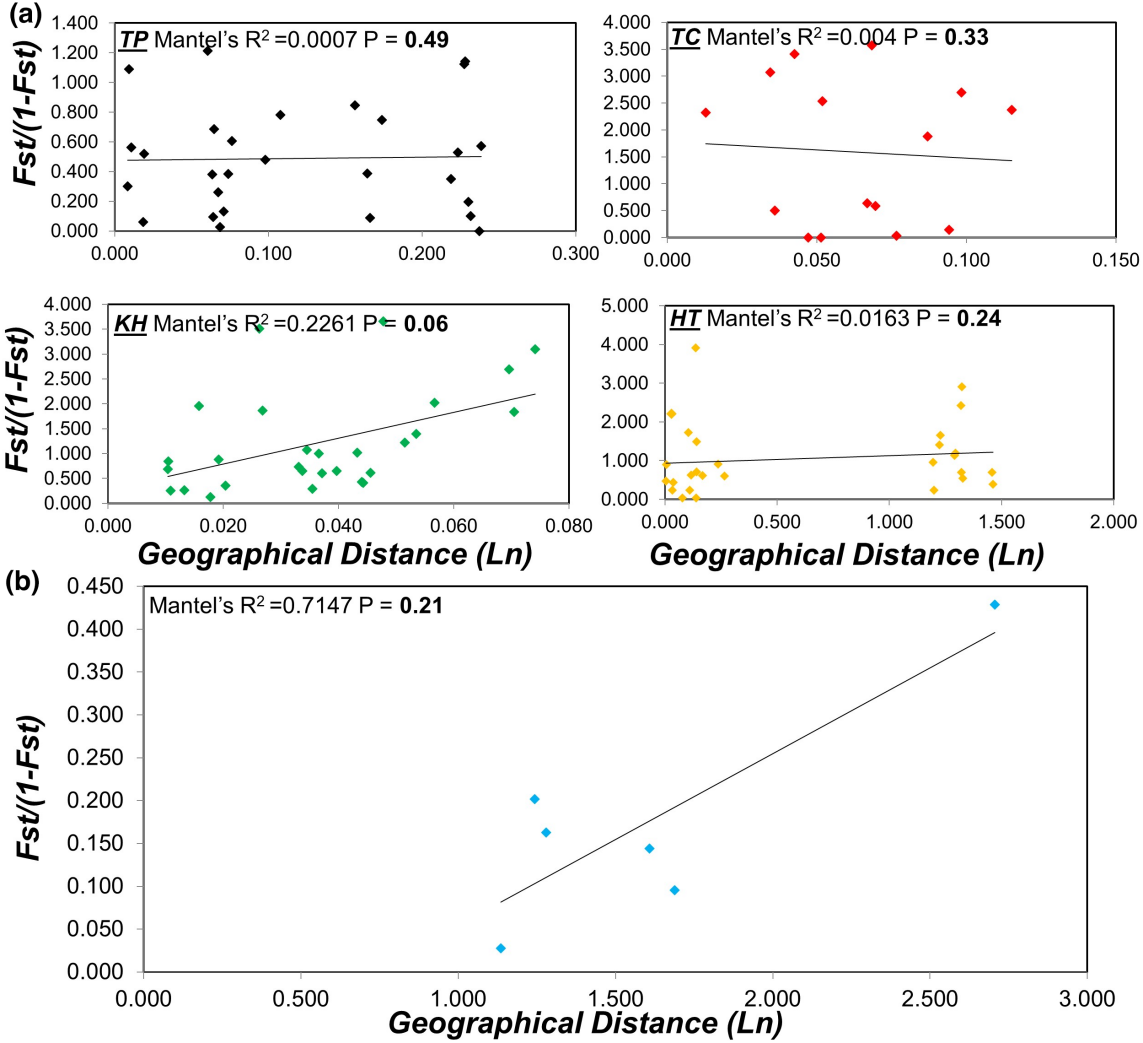
Correlations between genetic differentiation and geographical distances (isolation by distance) (a) among *Pheidole megacephala* colonies and (b) among Taiwanese regions.

Our AMOVA results (Table 2) revealed significant genetic structures at every hierarchical level; we observed 9% genetic variation among regions (*F*_RT_ = 0.085), 43% among colonies (*F*_SR_ = 0.474), and 48% within colonies (*F*_ST_ = 0.519). The pairwise *F*_ST_ values were moderate but significant across all combinations (Appendix S4 and Table S2), with the pairwise comparisons involving KH revealing high *F*_ST_ values. We observed no significant positive correlations between geographical distance and genetic differentiation among the colonies and regions (Figure 3). It is worth noting that the isolation by distance is marginally significant in KH. The isolation by distance was not detected at the island scale most likely due to low sample size of the sampling regions (Figure 3b).

**TABLE 2:** AMOVA for *Pheidole megacephala* in Taiwan based on microsatellite data.

Source	Df	MS	Percentage of variation (%)	*F* value	*p*
Among regions	3	38.701	9	*F*_RT_ = 0.085	.001
Among colonies	26	16.341	43	*F*_SR_ = 0.474	.001
Within colonies	450	1.058	48	*F*_ST_ = 0.519	.001
Total	479		100		

**FIGURE 4:**
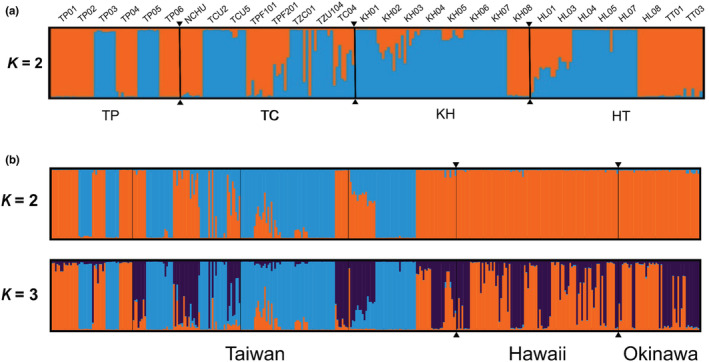
Population genetic structures based on Bayesian clustering analysis of *Pheidole megacephala* (a) among four Taiwanese regions (*K* = 2 for all sampled workers in all colonies) and (b) in Taiwan, Hawaii, and Okinawa (*K* = 2 and 3 for all sampled workers in all colonies).

Our Bayesian clustering analysis conducted through STRUCTURE revealed that the optimal partitioning of all colony samples was *K* = 2 (Figure 4). However, the population was not segregated into equivalent clusters on the basis of the four regions. The results indicated a weak population structure among the regions, suggesting a recent gene flow among these regions. As illustrated in Figure 4, most of the colonies sampled from TP and KH were present in clusters 1 (orange) and 2 (blue), respectively. The colonies sampled from TC and HT were partially observed in clusters 1 and 2 (Figure 4).

**FIGURE 5:**
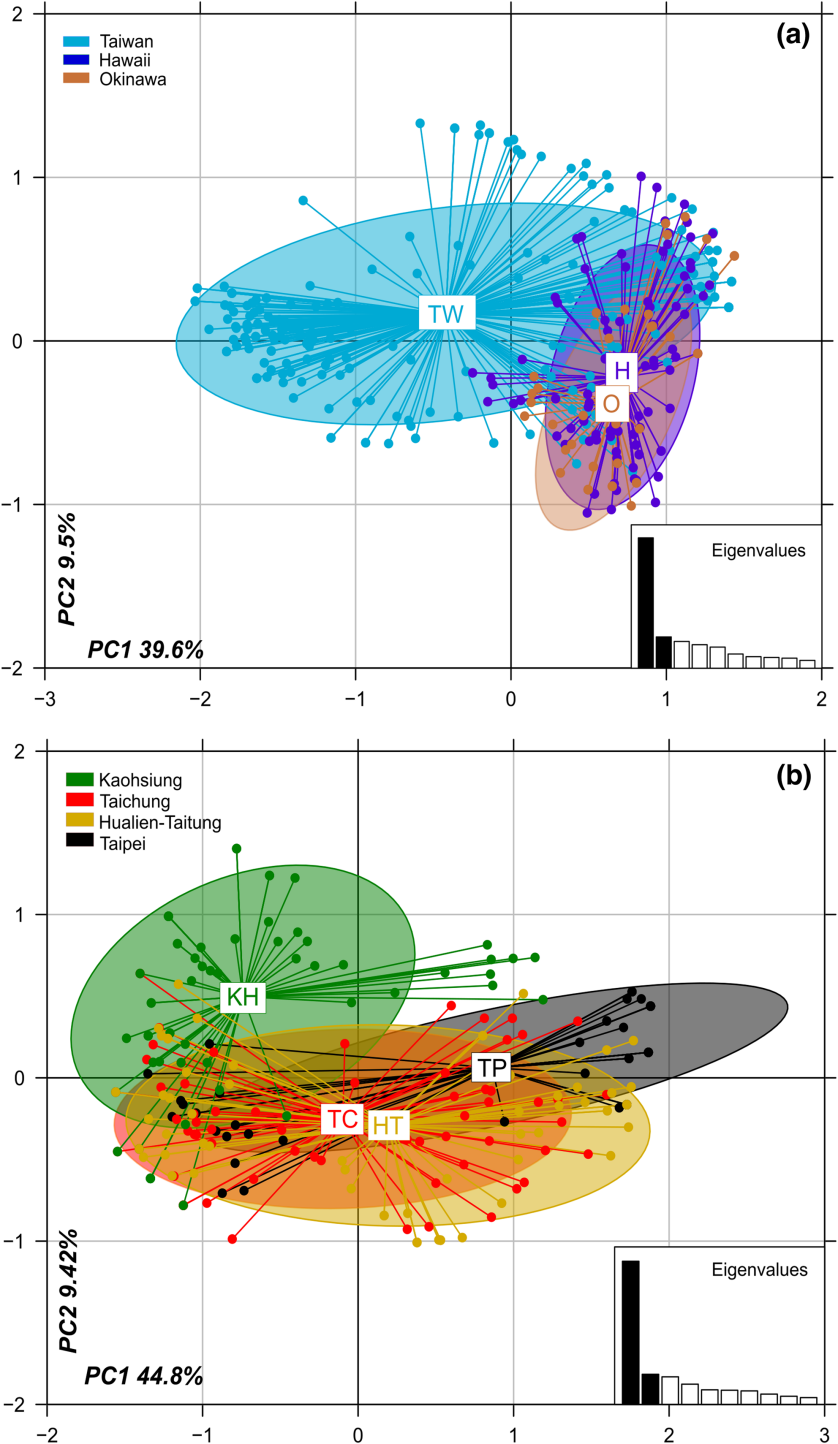
Principal component analysis clustering revealing the population structure across (a) four Taiwanese regions and (b) three Asia‐Pacific islands.

The PCA results revealed that the first two principal components accounted for 54.2% of the variation among Taiwan's four regions. The first principal component (44.8% of the total variance) mainly distinguished colonies in KH (southern Taiwan) from those in TP (northern Taiwan). The population structures of the colonies sampled from TC and HT, located in the central region of Taiwan, considerably overlapped with each other. Although the colonies sampled from KH and TP partially overlapped with those sampled from TC and HT, the second principal component (9.4% of the total variance) indicated that the colonies sampled from KH possessed unique genetic structures distinct from those of the colonies sampled from the central region (TC and HT; Figure 5). Regarding the population structure, the PCA results were noted to be consistent with the STRUCTURE results.

### 3.3 Genetic differentiation and population structures among the three islands

The pairwise *F*_ST_ estimates demonstrated that the genetic differentiation between Okinawa and Hawaii was low (*F*_ST_ = 0.072). However, the differentiation between Taiwan and Okinawa and that between Taiwan and Hawaii were moderate (*F*_ST_ = 0.176 and 0.177, respectively; Table 3).

**TABLE 3:** Pairwise genetic differentiation in the studied *Pheidole megacephala* populations in Taiwan, Hawaii, and Okinawa.

*F*_ST_	Taiwan	Hawaii	Okinawa
Taiwan	—	0.001	0.001
Hawaii	0.177	—	0.001
Okinawa	0.176	0.072	—

The PCA results revealed that the first two principal components accounted for 49.1% of the variation among islands (Figure 5). The first principal component (39.6% of the total variance) partially distinguished the populations in Hawaii and Okinawa from those in Taiwan. The Hawaiian and Okinawan populations were noted to almost overlap with each other, suggesting the genetic similarity between the two populations. The second principal component (9.5% of the total variance) could explain the genetic variation in the populations within the Okinawa and Hawaii regions. Our Bayesian clustering analysis revealed that the optimal partitioning of all colony samples was *K* = 2 (Figure 4). At *K* = 3, the populations from Hawaii and Okinawa exhibited similar genetic patterns. However, a specific genetic cluster was observed only in the Taiwanese population.

### 3.4 Phylogenetic relationships

**FIGURE 6:**
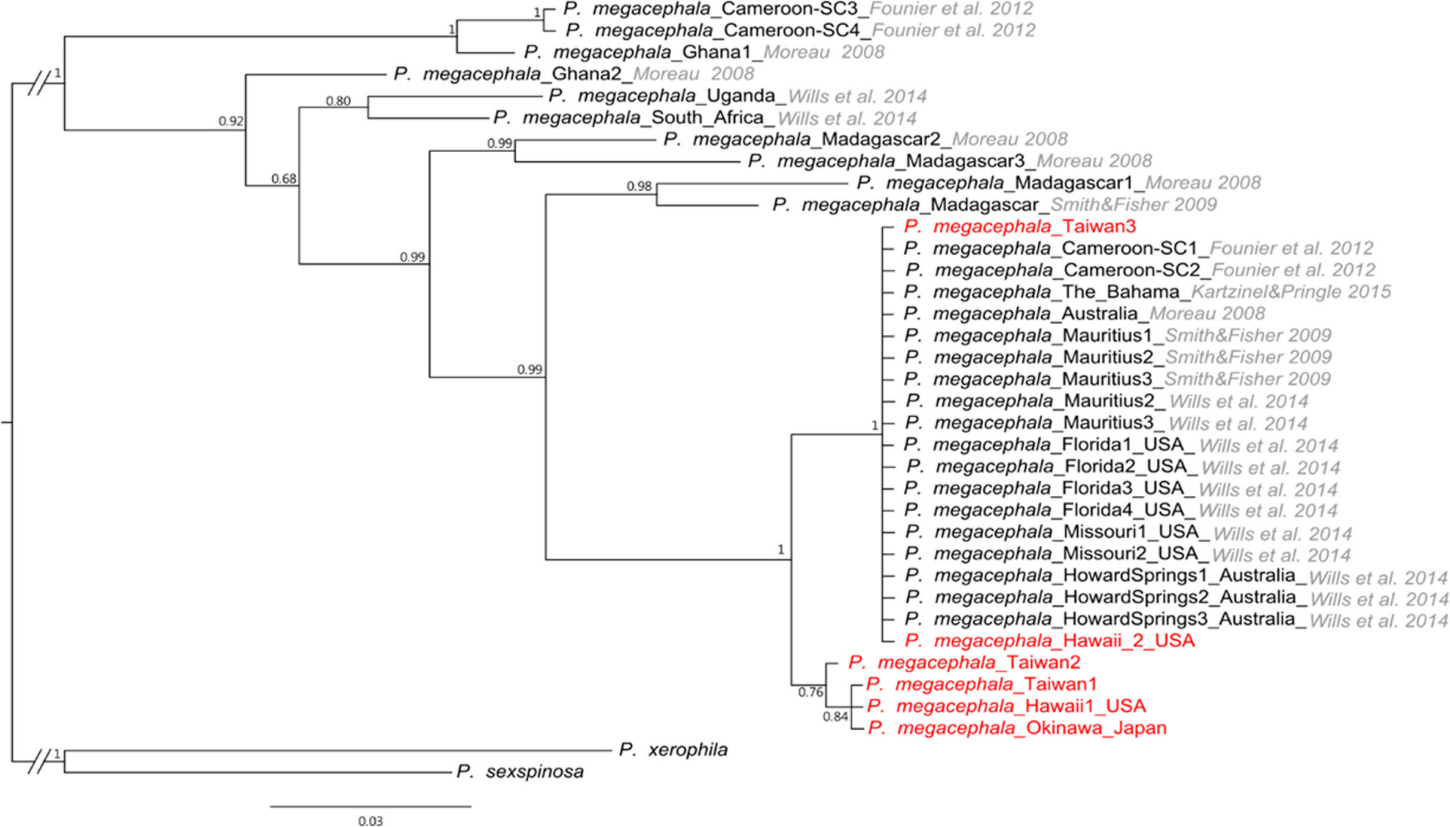
Bayesian inference tree based on 36 *COI* sequences (310 bp) of *Pheidole megacephala* from various localities with sister species—*Pheidole xerophila* and *Pheidole sexspinosa—*as outgroups. Red text denotes haplotypes identified in this study.

The phylogenetic tree (Figure 6)—constructed using 310 bp of the mitochondrial control region—revealed that the Taiwanese population could be separated into three major clusters: Taiwan 1 (TW1), 2 (TW2), and 3 (TW3). TW1 and TW2 were separated by one mutational step (0.3%), whereas TW1 and TW3 were separated by seven mutational steps (2.3%). TW2 and TW3 were separated by six mutational steps (1.9%). Moreover, TW1 was most common haplotype (representing 77% of the individuals analyzed), followed by TW3 and TW2 (representing 20% and 3% of the individuals analyzed, respectively). TW1 was prevalent throughout Taiwan, whereas TW3 was noted in TC, KH, and HT but not in TP. By contrast, TW2 was noted only in KH (Figure 1).

TW3, belonging to the same phylogenetic group, resembles haplotypes recovered from specimens collected from the United States (Missouri and Florida), Australia, Cameroon, Mauritius, and Caribbean countries, whereas TW1 resembles haplotypes recovered from specimens collected from Hawaii and Okinawa (Figure 6). By contrast, TW2 represents a novel haplotype previously unreported in other regions.

## 4 DISCUSSION

Our results demonstrate that *P. megacephala* in Taiwan has experienced a substantial reduction in genetic diversity. Comparisons of the genetic diversity of *P. megacephala* in Taiwan, Australia, and South Africa (Fournier et al., 2009) indicated that *A*r in Taiwan (2.615 ± 0.149) is lower than that in South Africa (6.129 ± 0.631) but similar to that in Australia (2.545 ± 0.226). Similar patterns of reduced genetic diversity have been commonly reported in the introduced ranges of most invasive ants (Fournier et al., 2005; Ross et al., 1993; Vogel et al., 2010) and are best explained by population bottlenecks resulting from small founding populations (Arca et al., 2015; Colautti et al., 2017; Kinziger et al., 2011; Sakai et al., 2001).

Although we observed a lower *A*r level in the *P. megacephala* populations in Taiwan than in those in South Africa, no evidence of heterozygosity excess was noted in the populations in Taiwan. The signature of heterozygosity excess is detectable in a bottlenecked population because the loss of alleles is typically faster than gene diversity during bottlenecks (Hedrick et al., 1986). The heterozygosity excess method, however, captures only relatively recent bottlenecks occurring less than 0.2–4.0 adequate population size (*N*e) generations ago (Luikart & Cornuet, 1998). Therefore, our finding likely reflects that the *P. megacephala* bottleneck during its introduction to Taiwan was not recent (Luikart et al., 1998). Reduced genetic diversity without a signature of heterozygosity excess was also observed in another introduced *P. megacephala* population of a similar age in Australia (Fournier et al., 2009). The heterozygosity excess method may detect a mutation–drift equilibrium in two introduced populations; however, its ability to detect a bottleneck can be affected by other factors (e.g., number of loci and population size), in addition to population recovery (Zepeda‐Paulo et al., 2016).

Our analyses of Taiwan's *P. megacephala* population demonstrated that the levels of genetic differentiation among regions were significant but low (except in KH). We also observed no significant positive relationship between geographical and genetic distances (isolation by distance). Our finding corroborates those of most previous studies that have reported limited isolation by distance in the ant's introduced ranges. For example, a study observed no correlation between genetic and geographical distances of up to 3000 km in Australia, which was engendered by a high level of gene flow between *P. megacephala* populations in human‐dominating habitats (Fournier et al., 2009). These results are consistent with the predictions of an urban facilitation model of gene flow in other globally introduced Argentine ant populations in the United States, where urbanization unites ant populations through human‐assisted long‐distance dispersal (Tsutsui & Case, 2001). Nevertheless, a systematic sampling scheme, including a line transect across the island, may aid in understanding the dispersal dynamics of *P. megacephala* in Taiwan.

The bridgehead effect is a major pathway facilitating global ant invasion. Bertelsmeier et al. (2018) demonstrated that most *P. megacephala* populations intercepted at ports in the United States and New Zealand originated from the species' initial invasive populations. Similarly, recent port interception records revealed that all intercepted *P. megacephala* populations in Taiwan originated from a nonnative region (Lee, Weng, et al., 2020). As the spread of *P. megacephala* coincides with the first historical waves of globalization (Bertelsmeier et al., 2017), we speculated that the spread of *P. megacephala* to Taiwan might be linked to the historical trade openness during the Qing dynasty's colonization, during which four treaty ports—Keelung and Tamsui located in northern Taiwan, and Anping and Takow located in southern Taiwan—were opened to global trade. After 1860, the volume of good trades increased considerably (Yuju, 2017). To date, the Kaohsiung and Keelung ports are Taiwan's international commerce centers. This speculation is supported by our STRUCTURE results that the current distribution of *P. megacephala* may have resulted from two separate introduction events, one in TP (northern Taiwan) and the other in KH (southern Taiwan), both of which were most likely introduced from the ant's nonnative regions. Moreover, this speculation is supported by our phylogenetic analysis of *P. megacephala* collections from Taiwan compared with other populations from native and exotic ranges, suggesting two or three possible lineages.

Haplotype TW1, which shares a common origin with introduced populations in Okinawa and Hawaii, was responsible for the region‐wide distribution in Taiwan. These *P. megacephala* populations may have arisen multiple times from unknown origins, considering that research on the phylogenetics of the test species is limited. Additionally, the PCA and STRUCTURE results indicate an apparent nuclear genetic similarity among the three *P. megacephala* populations. All of these results suggest a high level of connectivity among the three islands and that the interisland spread of *P. megacephala* might have been expected. This suggestion is supported by the extensive human immigration events occurring in Japan, Taiwan (i.e., Okinawa and Taiwan; Taiwan was under Japanese rule during the period under consideration), and Hawaii during the late 19th and early 20th centuries (Boyd, 1971; Matsumoto, 1982). Considering the ant's hitchhiker‐like nature, we speculated that *P. megacephala* might have been frequently transported among these regions through these immigration events. A similar genetic pattern was also reported for another invasive ant, *Anoplolepis gracilipes* (yellow crazy ant), in southern Japan, Taiwan, and Hawaii (Lee, Lin, et al., 2020), reinforcing the role of human‐assisted jump dispersal in shaping the genetic structure of invasive ants.

We observed a previously unreported haplotype, namely TW2. TW2 was limited to KH and was identified in a park 3 km from an international seaport (Port of Kaohsiung). In 2018, 100 colonies of imported red fire ants were discovered in the container yard at the Port of Kaohsiung (received cargo from China (60%) and the United States (40%); Wylie et al., 2020). The port of Kaohsiung is one of the busiest and largest seaports in the world, ranking 15th for international container shipping (Lauriat, 2019; Niimi, 2004). The low occurrence of TW2 (12.5%) and its limited distribution in KH suggest that the introduction of individuals harboring this haplotype occurred recently. Additional comprehensive studies on *P. megacephala* populations from other Asia‐Pacific regions are necessary to clarify their invasion history.

## AUTHOR CONTRIBUTIONS

**Kuan‐Ling Liu:** Conceptualization (supporting); formal analysis (lead); investigation (lead); visualization (lead); writing – original draft (lead); writing – review and editing (equal). **Shu‐Ping Tseng:** Methodology (equal); writing – review and editing (equal). **Haruki Tatsuta:** Resources (supporting); writing – review and editing (equal). **Kazuki Tsuji:** Resources (supporting); writing – review and editing (equal). **Jia‐Wei Tay:** Resources (supporting); writing – review and editing (supporting). **G. Veera Singham:** Validation (equal); writing – review and editing (equal). **Chin‐Cheng Scotty Yang:** Conceptualization (supporting); methodology (supporting); validation (equal); writing – review and editing (equal). **Kok Boon Neoh:** Conceptualization (supporting); funding acquisition (lead); supervision (lead); writing – original draft (lead); writing – review and editing (equal).

## CONFLICT OF INTEREST

The authors declare no competing financial interests.

## Supporting information


Appendix S1.

Appendix S2.

Appendix S3.

Appendix S4.

Appendix S5.

## Data Availability

All raw sequence files have been deposited in the National Center for Biotechnology Information (NCBI) under GenBank accession numbers ON524410‐ON524414 and ON528936.
