# Supplementary material for: Population genetic structure of the globally introduced big‐headed ant in Taiwan
Source: Ecol Evol. 2022 Dec 23;12(12):e9660. doi: 10.1002/ece3.9660 (PMC9789323; doi:10.1002/ece3.9660)
Supplement: Supplementary file 1 — Appendix S1. [file ECE3-12-e9660-s005.docx]

Supplementary file 1

Site collection of *Pheidole megacephala*

| Country | City/ State | Coordinate |
| --- | --- | --- |
| Taiwan | Taipei (TP) |  |
|  | TP01 | 121.534324, 25.099249 |
|  | TP02 | 121.582990, 25.082667 |
|  | TP03 | 121.515758, 25.070323 |
|  | TP04 | 121.510334, 25.058625 |
|  | TP05 | 121.559944, 24.987073 |
|  | TP06 | 121.534924, 25.032420 |
|  | Taichung (TC) |  |
|  | NCHU01 | 120.677443, 24.118872 |
|  | TC04 | 120.730310, 24.148561 |
|  | TCU2 | 120.626001, 24.341050 |
|  | TCU5 | 120.624818, 24.350140 |
|  | TPF101 | 120.741127, 24.149892 |
|  | TPF201 | 120.748707, 24.146398 |
|  | TZC01 | 120.731028, 24.212496 |
|  | TZU104 | 120.712017, 24.210676 |
|  | Kaohsiung (KH) |  |
|  | KH01 | 120.300133, 22.682507 |
|  | KH02 | 120.305394, 22.646307 |
|  | KH03 | 120.294894, 22.649175 |
|  | KH04 | 120.313852, 22.614296 |
|  | KH05 | 120.324176, 22.616242 |
|  | KH06 | 120.286275, 22.639150 |
|  | KH07 | 120.293212, 22.631445 |
|  | KH08 | 120.339560, 22.619787 |
|  | Hualien and Taitung (HT) |  |
|  | HL01 | 121.644378, 24.131259 |
|  | HL03 | 121.624870, 23.993263 |
|  | HL04 | 121.619320, 23.992730 |
|  | HL05 | 121.594475, 23.971070 |
|  | HL07 | 121.534041, 23.921358 |
|  | HL08 | 121.525525, 23.893124 |
|  | TT01 | 121.144555, 22.761696 |
|  | TT03 | 121.143593, 22.756942 |
| Japan | Okinawa | Undisclosed |
| U.S.A. | Hawaii |  |
|  | H1 | -157.786880, 21.267487 |
|  | H2 | -157.811446, 21.295630 |
|  | H3 | -157.819931, 21.269029 |
|  | H4 | -157.822462, 21.268863 |
|  | H5 | -157.777397, 21.269291 |
|  | H6 | -158.095522, 21.611056 |
|  | H7 | -158.089518, 21.538646 |
|  | H8 | -157.844857, 21.301290 |
|  | H9 | -157.859821, 21.305125 |
|  | H10 | -157.816851, 21.300039 |
|  | H11 | -157.724717, 21.395420 |
|  | H12 | -158.000315, 21.385804 |
