# Supplementary material for: Population genetic structure of the globally introduced big‐headed ant in Taiwan
Source: Ecol Evol. 2022 Dec 23;12(12):e9660. doi: 10.1002/ece3.9660 (PMC9789323; doi:10.1002/ece3.9660)
Supplement: Supplementary file 2 — Appendix S2. [file ECE3-12-e9660-s001.docx]

Supplementary file 2

**Materials and methods**

**Ant Identification**

*Pheidole megacephala* was identified based on the main characteristic of the postpetiole with a posterodorsal and anteroventral bulge(Bolton 1994; Lin 1998; Sarnat et al. 2015).

**Scanning Electron Microscope (SEM) observation**

Three workers per colony were used to observe the hairs on ant’s petiole under scanning electron microscope (ABT-150S, Topcon; Tokyo, Japan); samples have air-dried and coated with gold using an ion sputter (Polaron SC502, Fisons; Ipswich, United Kingdom).

**Results**

Cryptic species found in urban areas have shorter spines on the propodeum with hairs on the petiole end in a point; while that rainforest populations with brush from Fournier et al. (2012). Either pointed hair or brush hair present in samples between urban and forest area. By contrast, morphological observation under SEM hairs end in multiple splits (3-6 splits) were consistently observed, indicating absence of cryptic species (Fig.1).


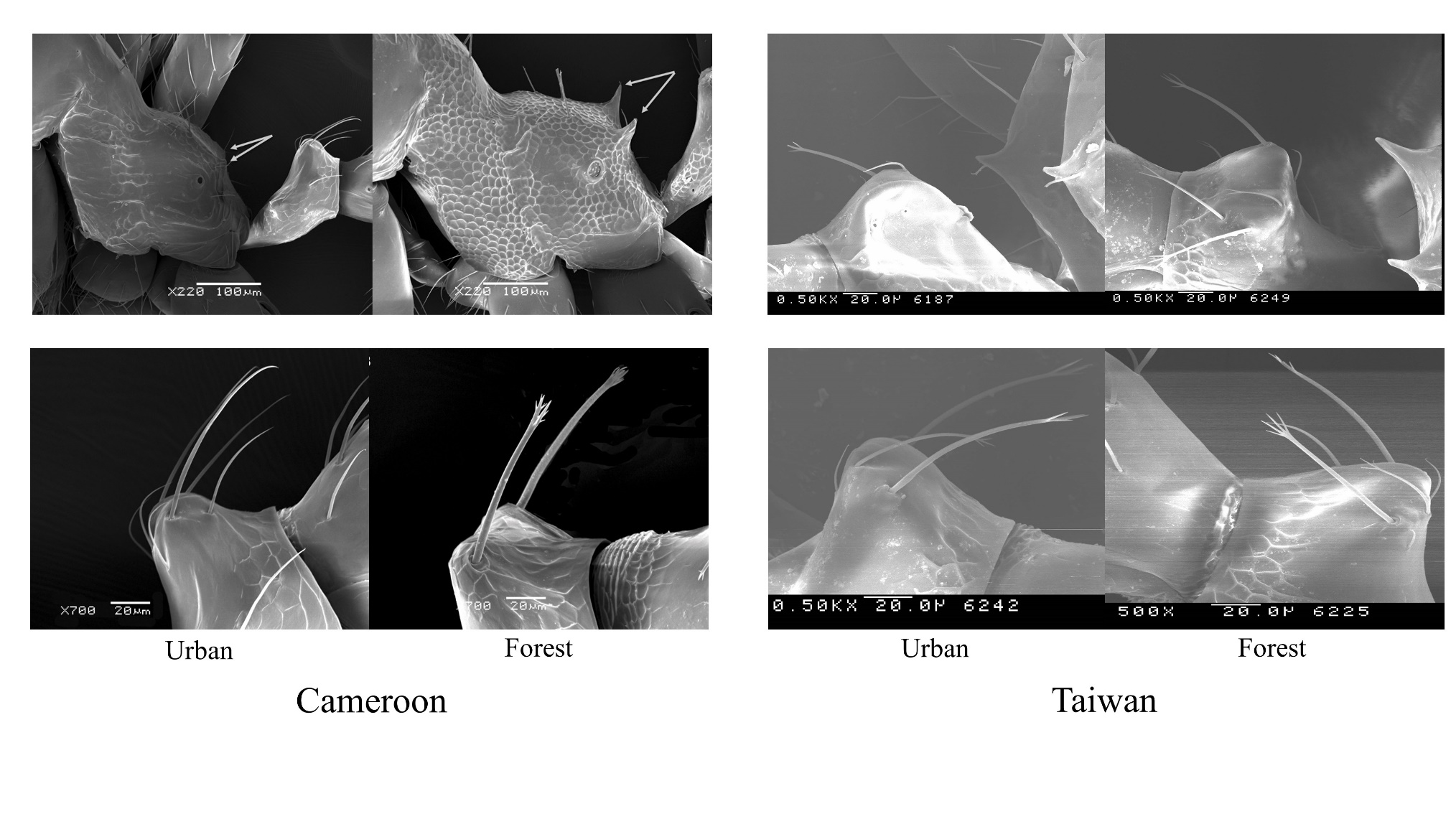


Figure.1 Comparison between *Pheidole megacephala* in Taiwan (present study) and Cameroon Fournier et al. (2012).Views of hairs on the petiole and spines on the propodeum of ant in urban and forest area. Magnification and scale bar are indicated for each scanning electron microscopic image.

**Reference**

Bolton B (1994) Identification guide to the ant genera of the world. Harvard University Press,

Fournier D, Tindo M, Kenne M, Masse PSM, Van Bossche V, De Coninck E, Aron S (2012) Genetic structure, nestmate recognition and behaviour of two cryptic species of the invasive big-headed ant Pheidole megacephala PLoS One 7:e31480

Lin CC (1998) Systematic and zoogeographic studies on the ant subfamily Myrmicinae in Taiwan (Hymenoptera: Formicidae). Ph. D. Dissertation, National Taiwan University Press, Taiwan

Sarnat EM, Fischer G, Guénard B, Economo EPJZ (2015) Introduced Pheidole of the world: taxonomy, biology and distribution:1
