# Supplementary material for: Population genetic structure of the globally introduced big‐headed ant in Taiwan
Source: Ecol Evol. 2022 Dec 23;12(12):e9660. doi: 10.1002/ece3.9660 (PMC9789323; doi:10.1002/ece3.9660)
Supplement: Supplementary file 3 — Appendix S3. [file ECE3-12-e9660-s003.docx]

Supplementary file 3

Specimens used, their locality and GenBank accession numbers for COI sequences

| Species | Accession no. | Locality | Reference | Note |
| --- | --- | --- | --- | --- |
| *Pheidole megacephala* | KF171406 | Howard Springs, Australia | (Wills et al. 2014) |  |
|  | KF171407 | Howard Springs, Australia | (Wills et al. 2014) |  |
|  | KF171408 | Howard Springs, Australia | (Wills et al. 2014) |  |
|  | EF518366 | Australia | (Moreau 2008) |  |
|  | KF171417 | Missouri, USA | (Wills et al. 2014) |  |
|  | KF171418 | Missouri, USA | (Wills et al. 2014) |  |
|  | KF171392 | Florida, USA | (Wills et al. 2014) |  |
|  | KF171393 | Florida, USA | (Wills et al. 2014) |  |
|  | KF171394 | Florida, USA | (Wills et al. 2014) |  |
|  | KF171395 | Florida, USA | (Wills et al. 2014) |  |
|  | KF171410 | Mauritius | (Wills et al. 2014) |  |
|  | KF170411 | Mauritius | (Wills et al. 2014) |  |
|  | EF610023 | Mauritius | (Smith and Fisher 2009) |  |
|  | EU150294 | Mauritius | (Smith and Fisher 2009) |  |
|  | EU150291 | Mauritius | (Smith and Fisher 2009) |  |
|  | KP253849 | The Bahamas | (Kartzinel and Pringle 2015) |  |
|  | EF518412 | Madagascar | (Moreau 2008) |  |
|  | EF518425 | Madagascar | (Moreau 2008) |  |
|  | EF518419 | Madagascar | (Moreau 2008) |  |
|  | HQ54739 | Madagascar | (Smith and Fisher 2009) |  |
|  | EF518413 | Ghana | (Moreau 2008) |  |
|  | EF518421 | Ghana | (Moreau 2008) |  |
|  | KF171422 | Uganda | (Wills et al. 2014) |  |
|  | KF171421 | South Africa | (Wills et al. 2014) |  |
|  | NA | Cameroon | (Fournier et al. 2012) | Personal communication |
|  | NA | Cameroon | (Fournier et al. 2012) | Personal communication |
|  | NA | Cameroon | (Fournier et al. 2012) | Personal communication |
|  | NA | Cameroon | (Fournier et al. 2012) | Personal communication |
| *Pheidole xerophila* | EF518445 | NA | (Moreau 2008) |  |
| *Pheidole sexspinosa* | EF518404 | Palau | (Moreau 2008) |  |

Fournier D, Tindo M, Kenne M, Masse PSM, Van Bossche V, De Coninck E, Aron S (2012) Genetic structure, nestmate recognition and behaviour of two cryptic species of the invasive big-headed ant Pheidole megacephala PLoS One 7:e31480

Kartzinel TR, Pringle RM (2015) Molecular detection of invertebrate prey in vertebrate diets: trophic ecology of Caribbean island lizards Molecular Ecology Resources 15:903-914 doi:10.1111/1755-0998.12366

Moreau CS (2008) Unraveling the evolutionary history of the hyperdiverse ant genus Pheidole (Hymenoptera: Formicidae) Molecular Phylogenetics and Evolution 48:224-239 doi:<https://doi.org/10.1016/j.ympev.2008.02.020>

Smith MA, Fisher BL (2009) Invasions, DNA barcodes, and rapid biodiversity assessment using ants of Mauritius Frontiers in Zoology 6:31 doi:10.1186/1742-9994-6-31

Wills BD, Moreau CS, Wray BD, Hoffmann BD, Suarez AV (2014) Body size variation and caste ratios in geographically distinct populations of the invasive big-headed ant, Pheidole megacephala (Hymenoptera: Formicidae) Biological Journal of the Linnean Society 113:423-438 doi:10.1111/bij.12386
