# Supplementary material for: Population genetic structure of the globally introduced big‐headed ant in Taiwan
Source: Ecol Evol. 2022 Dec 23;12(12):e9660. doi: 10.1002/ece3.9660 (PMC9789323; doi:10.1002/ece3.9660)
Supplement: Supplementary file 4 — Appendix S4. [file ECE3-12-e9660-s002.docx]

Supplementary file 4

Table S1 Test for genetic bottleneck using two-phase model (TPM) with Wilcoxon test (two-tailed) in four administrative regions

| Population |  | TP | TC | KH | HT |
| --- | --- | --- | --- | --- | --- |
| Wilcoxon test | TPM | 0.938 | 0.813 | 0.813 | 1.000 |
|  | Model shift | Normal | Normal | Normal | Normal |

Table S2 Pairwise genetic differentiation among the four administrative regions studied *Pheidole megacephala* population in Taiwan

| *F*_ST_ \ *Nm* | TP | TC | KH | HT |
| --- | --- | --- | --- | --- |
| TP | - | 1.538 | 0.583 | 2.614 |
| TC | 0.140* | - | 1.729 | 9.093 |
| KH | 0.300* | 0.126* | - | 1.240 |
| HT | 0.087* | 0.027* | 0.168* | - |
