# Supplementary figures and images for: Population genetic structure of the globally introduced big‐headed ant in Taiwan
Source: Ecol Evol. 2022 Dec 23;12(12):e9660. doi: 10.1002/ece3.9660 (PMC9789323; doi:10.1002/ece3.9660)

Supplementary file 5


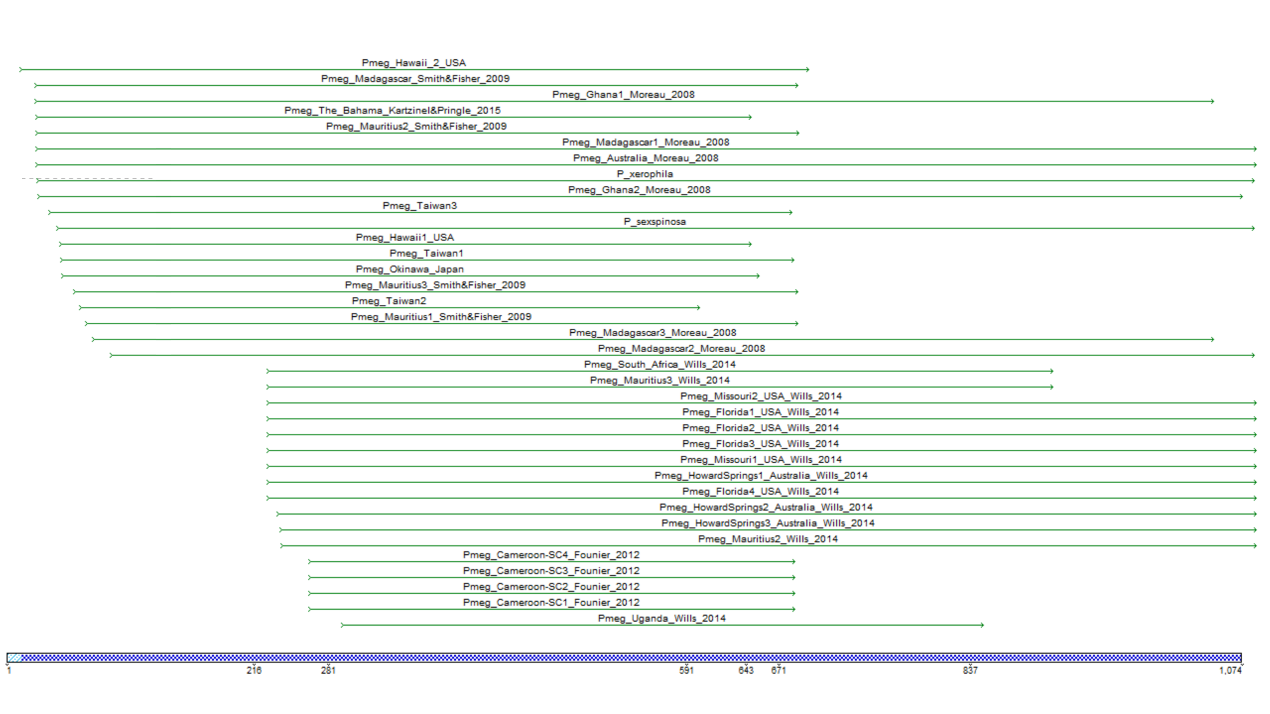
 Figure 1. The alignments of all sequences obtained in this study

Supplement: Supplementary file 5 — Appendix S5. [file ECE3-12-e9660-s004.docx]
